# Limb salvage surgery with joint preservation for malignant humeral bone tumors: operative procedures and clinical application

**DOI:** 10.1186/s12893-019-0519-3

**Published:** 2019-05-30

**Authors:** Jie Zhao, Ming Xu, Kai Zheng, Xiuchun Yu

**Affiliations:** 1 Department of Orthopedics, The PLA 960th Hospital of China, 25#, Shifan Road, Jinan, 250031 China; 2 First Clinical Medical College, Shandong University of Traditional Chinese Medicine, 4655#, Daxue Road, Jinan, 250355 China

**Keywords:** Joint preservation-limb salvage surgery-malignant humeral bone tumors-operative procedure

## Abstract

**Background:**

However, the application of limb salvage with joint preservation is controversial. The purpose of this study is to propose a selection strategy of joint-sparing operative procedures for humeral malignancies based on tumor origin, site and bone strength.

**Methods:**

The medical data of 28 patients with humeral malignancies treated at our institute from January 2010 to December 2016 were analyzed retrospectively. The patients had a median age of 51 years (range, 8–82 years). Bone strength scoring system was utilized to evaluated bone strength of the tumor. Four joint-sparing surgical methods were performed on selected patients. Evaluation of limb function was based on the Musculoskeletal Tumor Society scoring system. Two-sample t-test was used to compare patient group data such as bone strength score and postoperative Musculoskeletal Tumor Society score.

**Results:**

The mean follow-up period for the 7 patients with primary malignancies was 45 months (range, 15–66 months). One patient died due to recurrence and lung metastasis, while the remaining 6 patients (6/7, 85.7%) survived without recurrence. For the 21 patients with metastases, 5 survived with tumors, with an average survival time of 25.8 months (range, 9–48 months). The rest died from progression of the primary tumors. The mean bone strength score for the biological reconstruction group and non-biological reconstruction group was respectively 9.7 ± 1.3 and 12.9 ± 1.2. A significant difference between the 2 groups (*p <* 0.05) was found. Mean postoperative Musculoskeletal Tumor Society score was respectively 27.2 ± 1.8 and 26.1 ± 1.7 for the 2 groups. There was no significant difference between the 2 groups (*p* > 0.05). Non-oncological complications included fracture (1), aseptic loosening (1) and radial nerve injury (1).

**Conclusions:**

Alcohol devitalized autograft replantation is applicable for diaphyseal humeral primary malignancies, with a good response to chemotherapy and a low bone strength score (≤10). In situ microwave ablation is suitable for diaphyseal and (or) metaphyseal low-grade malignant bone tumors or metastases with a low bone strength score (≤10). Intercalary prosthetic reconstruction is preferred for diaphyseal metastases with a high bone strength score (> 10).

## Background

The humerus is the third most common site for the development of primary malignant bone tumors, and is the second most common site affected by metastatic bone tumors involving long bones [1, 2]. As recent advancement of radiation therapy and chemotherapy, limb salvage procedures have been the main method of surgical treatment for humeral malignancies. Nowadays, more than 80% of the patients are treated with limb salvage surgery without critically compromising oncological principles [3]. Most humeral malignant bone tumors are located on the epiphysis and/or the metaphysis. Wide resection of the tumors often requires sacrifice of the native shoulder joint based on the principle of treatment for malignant bone tumors. However, the ideal surgical procedure for reconstruction after proximal humeral resection remains controversial. The common reconstruction methods include osteoarticular allografts, allograft-prosthetic composites, endoprosthesis, clavicula pro humero, vascularized fibular grafts, extracorporeal inactivated autograft, or combined fibular graft with allograft/recycled autograft [4–10]. However, some humeral malignancies occur in the metaphysis or diaphysis, making it possible to preserve the adjacent joints.

However, there is no consistent application standards for various limb salvage operative procedures with joint preservation. We retrospectively reviewed medical records of 28 patients who had undergone joint-sparing limb salvage surgery for humeral malignant bone tumors at our institute. The purpose of this study is to propose a selection strategy of joint-preserving operative procedures for humeral malignant bone tumors based on tumor origin, site and bone strength.

## Methods

Inclusion criteria were as follows: (i) diaphysis or metaphysis humeral malignant bone tumors not involving major blood vessels and nerves; (ii) primary malignant bone tumors sensitive to neoadjuvant chemotherapy; (iii) life expectancy of patients with humeral metastases > 3 months; (iv) patients who underwent joint-sparing limb salvage surgery; (v) complete follow-up data. Exclusion criteria were as follows: (i) humeral malignant bone tumors involving the epiphysis; (ii) patients who underwent joint resection and reconstruction; (iii) incomplete follow-up data.

The clinical data of 28 eligible patients (12 males and 16 females) with humeral diaphysis or metaphysis malignant bone tumors treated at our institute between January 2010 and December 2016 were retrospectively analyzed (Table 1). The patients had a median age of 51 years (range, 8–82 years) at diagnosis. Pathological diagnosis of all patients was determined through aspiration biopsy. There were 7 patients with primary malignant bone tumors, 17 patients with metastatic bone tumors, and 4 patients with hematological malignancies. Apart from 3 chondrosarcoma patients, all patients with primary malignant bone tumors had received preoperative neoadjuvant chemotherapy. The chemotherapy regimens included cisplatin, doxorubicin and ifosfamide in accordance with a previously published report [11]. Joint-sparing limb salvage surgery was performed on patients who had an excellent response to chemotherapy. The degree of limb pain was judged using a visual analogue scale (VAS) and the patients were divided into mild, moderate and severe pain groups according to their VAS score.

**Table 1:** General information, bone strength score, surgical procedure, follow-up of 28 patients

Case	Diagnosis	site	bone strength score	Surgical procedure	Follow-up (months)	MSTS score	Complication	Outcomes
1	PNET	S5	10	AAR	28	24	Recurrence, lung metastasis	Dead
2	POS	S5	8	AAR	66	30		DFS
3	ES	S5	9	AAR	63	30		DFS
4	CS	S4	10	MA	54	29		DFS
5	CS	S4	9	MA	30	27		DFS
6	CS	S4	9	MA	60	27		DFS
7	ES	S45E1	12	MA	15	29		DFS
8	gastric cancer	S5	11	MA	15	27		Dead
9	Bladder cancer	S5	8	MA	15	26		Dead
10	Lung tumor cancer	S5	9	MA	6	27		Dead
11	kidney cancer	S4	9	MA	18	26		Dead
12	MM	S45	11	MA	12	26		SWT
13	NHL	S45	11	MA	18	26		Dead
14	Lung tumor cancer	S5	11	PF	18	26		Dead
15	Breast cancer	S5	14	PF	21	27		Dead
16	MM	S45	15	PF	9	25		SWT
17	MM	S5	12	PF	42	24	Fracture of internal fixation	Dead
18	Esophageal cancer	S5	14	INF	9	27		Dead
19	kidney cancer	S5	12	INF	24	25		Dead
20	Breast cancer	S5	12	IP	18	22	aseptic loosening	TBS
21	Liver cancer	S5	14	IP	6	28		Dead
22	Lung cancer	S5	14	IP	6	26	Radial nerve injury	Dead
23	Breast cancer	S5	14	IP	42	27		SWT
24	Lung cancer	S5	13	IP	6	29		Dead
25	Thyroid cancer	S5	13	IP	48	27		SWT
26	Lung cancer	S5	12	IP	6	26		Dead
27	kidney cancer	S5	12	IP	23	26		Dead
28	Lung cancer	S5	11	IP	12	27		Dead

*PNET* primitive neuroectodermal tumor, *POS* parosteal osteosarcoma, *ES* Ewing sarcoma, *CS* chondrosarcoma, *MM* multiple myeloma, *NHL* non-Hodgkin lymphoma, *AAR* alcohol inactivated autograft replantation, *MA* microwave ablation, *PF* plate fixation with bone cement, *INF* intramedullary nail fixation with bone cement, *IP* intercalary prosthesis, *DFS* disease-free survival, *SWT* survival with tumor

The preoperative imaging data of all patients were comprehensively analyzed to assess tumor site, nature and bone strength. Tumor boundaries were identified using magnetic resonance imaging (MRI) data. The tumors were in the diaphysis and/or metaphysis, and did not invade beyond the epiphyseal line or plate. According to the Musculoskeletal Tumour Society (MSTS) resection classification system [12], the defects were classified as S4 (4 patients), S5 (20 patients), S45 (3 patients) and S45E1 (one patient). Based on Mirel’s scoring system [13] and the invasion score for evaluating the extent of bone destruction through osteosarcoma [14], we made some improvements and proposed the bone strength scoring system. Five variables, including pain degree, tumor site, nature, length and transverse diameter, were utilized to evaluated the bone strength of the tumor segment (Table 2). The length and diameter of the tumors were classified as < 1/3, ≥1/3 and ≤ 2/3, > 2/3 of the length and diameter of the humeral diaphysis, respectively. Each variable was given 3 points, with a maximum score of 15 points. The higher the score, the lower the bone strength.

**Table 2:** Bone strength scoring system

Variable		Score	
	1	2	3
Pain	Mild	Moderate	Severe
site	Distal humerus	Diaphysis	Proximal humerus
Nature	Osteogenic	Mixed	Osteolytic
Length	< 1/3	≥1/3,≤2/3	> 2/3
Transverse diameter	< 1/3	≥1/3,≤2/3	> 2/3

All patients underwent limb salvage surgery with joint preservation. In accordance with tumor origin, site and bone strength score, four different surgical methods with joint-sparing were performed in selected patients. As shown in Tables 1, 13 patients underwent biological reconstruction while 15 patients underwent non-biological reconstruction. Biological reconstruction methods included alcohol inactivated autograft replantation (AAR) with joint preservation (3) and in situ microwave ablation (MA) (10). Non-biological reconstruction methods included intercalary prosthetic reconstruction (9) and intramedullary nail or steel plate osteosynthesis with adjunctive bone cement (6).

Routine postoperative follow-up was performed on all patients. Oncological outcome, functional results and imaging findings were assessed and recorded at every follow-up visit. Evaluation of limb function was based on the Musculoskeletal Tumor Society (MSTS) scoring system for the upper extremity [15] (Table 1).

SPSS software (version 17.0, IBM, Armonk, NY, USA) was used to perform statistical analyses. We used chi-square test to compare count data such as gender. Measurement data are presented as mean ± standard deviation. The measurement data were treated with normality test. Mann-Whitney U test was used to compare age difference which not conformed to normal distribution. Bone strengh score and postoperative MSTS score were accorded with the normal distribution. Two-sample T-test was used to compare them between the biological reconstruction group and non-biological reconstruction group. A two-tailed *P* value of < 0.05 was considered as statistically significant.

The research has been performed in accordance with the declaration of Helsinki. Our study was approved by the Ethics Committee of the PLA 960th hospital. All adult patients and parents for children who participate in the study provided written informed consent.

## Results

Postoperative follow-up was conducted on all patients. The mean follow-up duration for the 7 patients with primary malignant bone tumors was 45 months (range, 15–66 months). One patient (1/7, 14.3%) who had undergone alcohol inactivated autograft replantation had local recurrence 7 months after the initial operation, and eventually died of lung metastasis. The remaining 6 patients who had a primary malignant bone tumor were alive and tumor-free. 5 of the 21 patients with metastatic or hematological malignancies were alive with the tumor, with an average survival period of 25.8 months (range, 9-48 months). The remaining 16 patients died of tumor progression by the end of the follow-up period.

There was no significant difference in gender and age between the two groups (*p* > 0.05).The average bone strength score of patients included in the current study was 11.4 ± 2.0 (range, 8–15). The mean bone strength score of the biological reconstruction group and non-biological reconstruction group was 9.7 ± 1.3 and 12.9 ± 1.2, respectively. There was significant statistical difference for bone strength score between the two groups (*p* < 0.05, Table 3).

**Table 3:** Comparisons between biological reconstruction and non-biological reconstruction

Comparative features	biological reconstruction	non-biological reconstruction	P value
Gender(F/M)	7 /6	9/6	1.000
Age(y)	56 ± 16.8	59 ± 10.6	0.289
Bone strength score	9.7 ± 1.3	12.9 ± 1.2	0.000
MSTS score	27.2 ± 1.8	26.1 ± 1.7	1.097

*MSTS* Musculoskeletal Tumor Society

*p* < 0.05 indicated a significant difference between the two groups

The mean postoperative MSTS score of the 28 patients was 26.6 ± 1.8 (range, 22–30). Postoperative limb function was able to meet the daily needs of the patients. The average MSTS score of the biological reconstruction group and non-biological reconstruction group was 27.2 ± 1.8 and 26.1 ± 1.7, respectively. There was no significant statistical difference for mean postoperative MSTS score between the two groups (*p* > 0.05, Table 3).

**Fig. 1:**
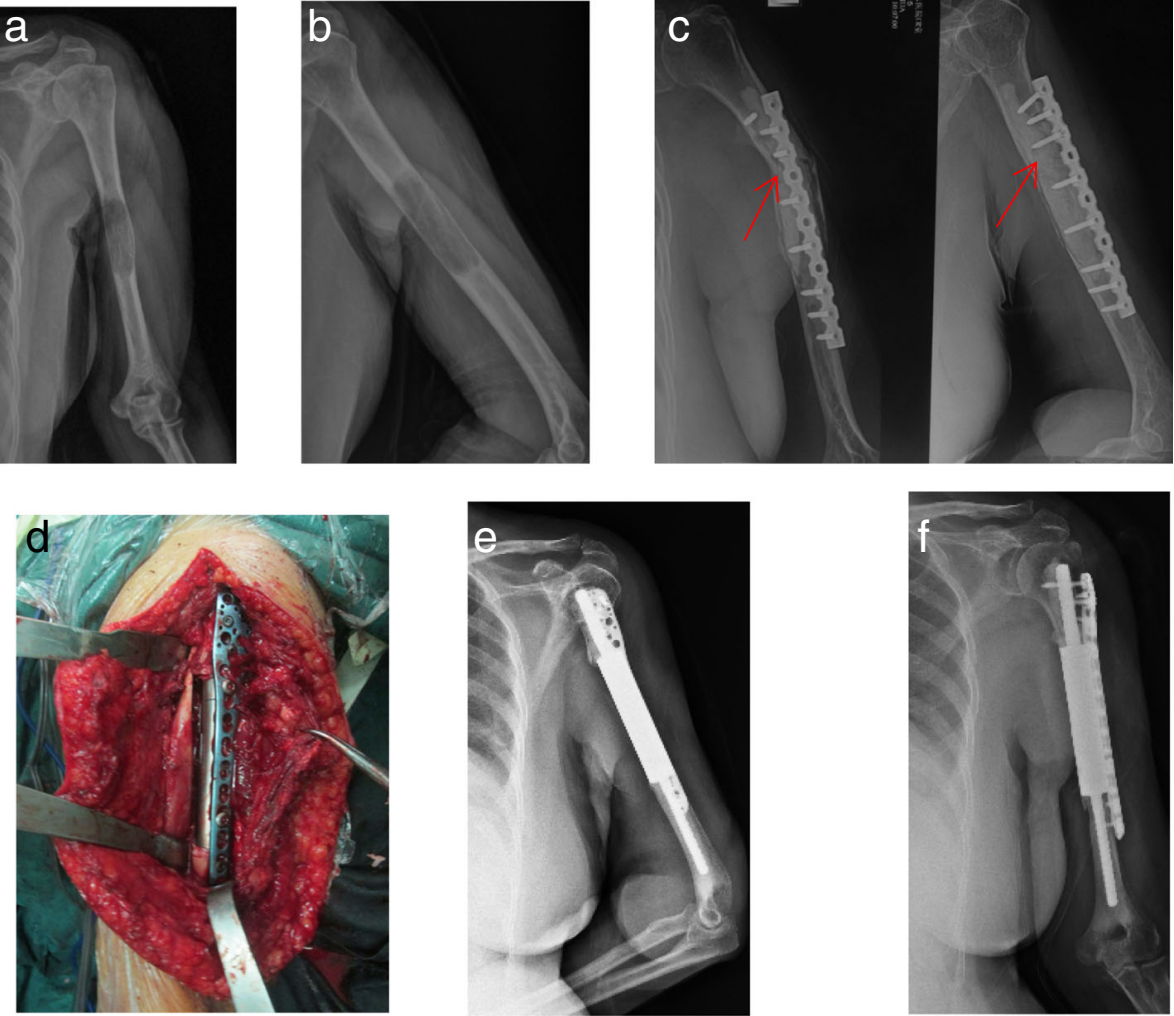
Case 20, a 62-y old female patient with osteolytic lesion due to multiple myeloma in the left humerus. The preoperative bone strength score is 12. **a, b** The tumor was located in the left humerus shaft (S5). **c** The patient underwent resection of the tumor and steel plate osteosynthesis with adjunctive bone cement. Fracture and screw breakage occurred 33 months after surgery (arrow). **d** Then, this patient underwent removement of the internal fixation and intercalary prosthetic reconstruction with an additional plate. **e, f** Postoperative X-ray indicated that intercalary prosthesis was in excellent position

**Fig. 2:**
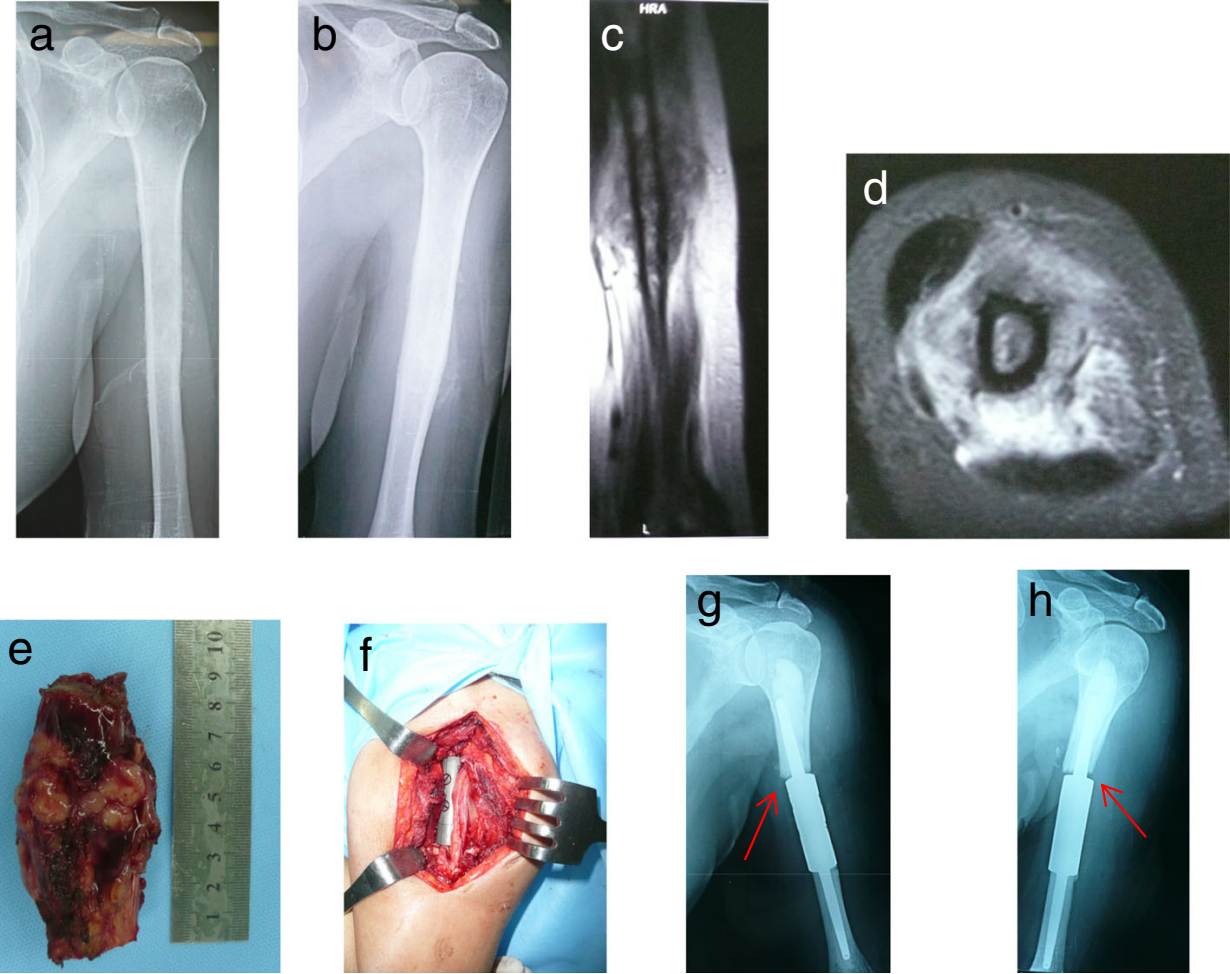
Case 23, a 65-y old female patient with metastatic tumor secondary to breast cancer. The bone strength score is 12. **a, b** The preoperative X-ray showed that the osteolytic destruction was located in the diaphysis of left humerus(S5 region). **c, d** The preoperative MRI indicated that the intramedullary destructive signals and soft-tissue swelling. **e, f** This patient underwent resection of the tumor and intercalary prosthetic reconstruction. **g, h** The proximal stem of the prosthesis occurred aseptic loosening 3 months after operation(arrow)

Non-oncological complications were encountered in 3 of the 28 patients (3/28, 10.7%), and included fracture and plate breakage in one patient (3.6%), aseptic loosening in another (3.6%), and radial nerve injury in yet another (3.6%). The patient with fracture and plate breakage was treated by removal of internal fixation and reconstruction of bone defect with intercalary prosthesis (Fig. 1). The patient with radial nerve injury was able to return to normal function after nerve electric stimulation treatment. The patient with aseptic loosening of segmental prosthesis received conservative treatment (Fig. 2).

## Discussion

The humerus is one of the most common sites of primary bone sarcomas and metastasis. At present, limb salvage has become the main surgical approach for the treatment of humeral malignant bone tumors, but the optimal procedure for preserving the adjacent shoulder and elbow joints remain a subject of debate. San-Julian et al. were the first to divide juxtarticular osteosarcoma in children into three types based on the relationship between the tumor border and the epiphyseal plate [16]. We once reported of the clinical application of alcohol-inactivated autograft replantation with articulation preservation for San-Julian type I osteosarcoma of the distal femur [11]. However, joint preservation for San-Julian type II tumors is still controversial. Some researchers believe that a safe margin cannot be obtained in such a situation [17]. By analyzing the impacts of tumor location, nature and bone destruction on a selection of limb salvage procedures, we previously found that biological reconstruction methods with joint preservation are feasible for diaphysis or metaphysis sarcomas with a low invasion score (≤8) [14]. Based on a comprehensive evaluation of tumor origin, site and bone strength in this study, we put forward a selection strategy of joint-preserving limb salvage surgery for malignant humeral bone tumors.

**Fig. 3:**
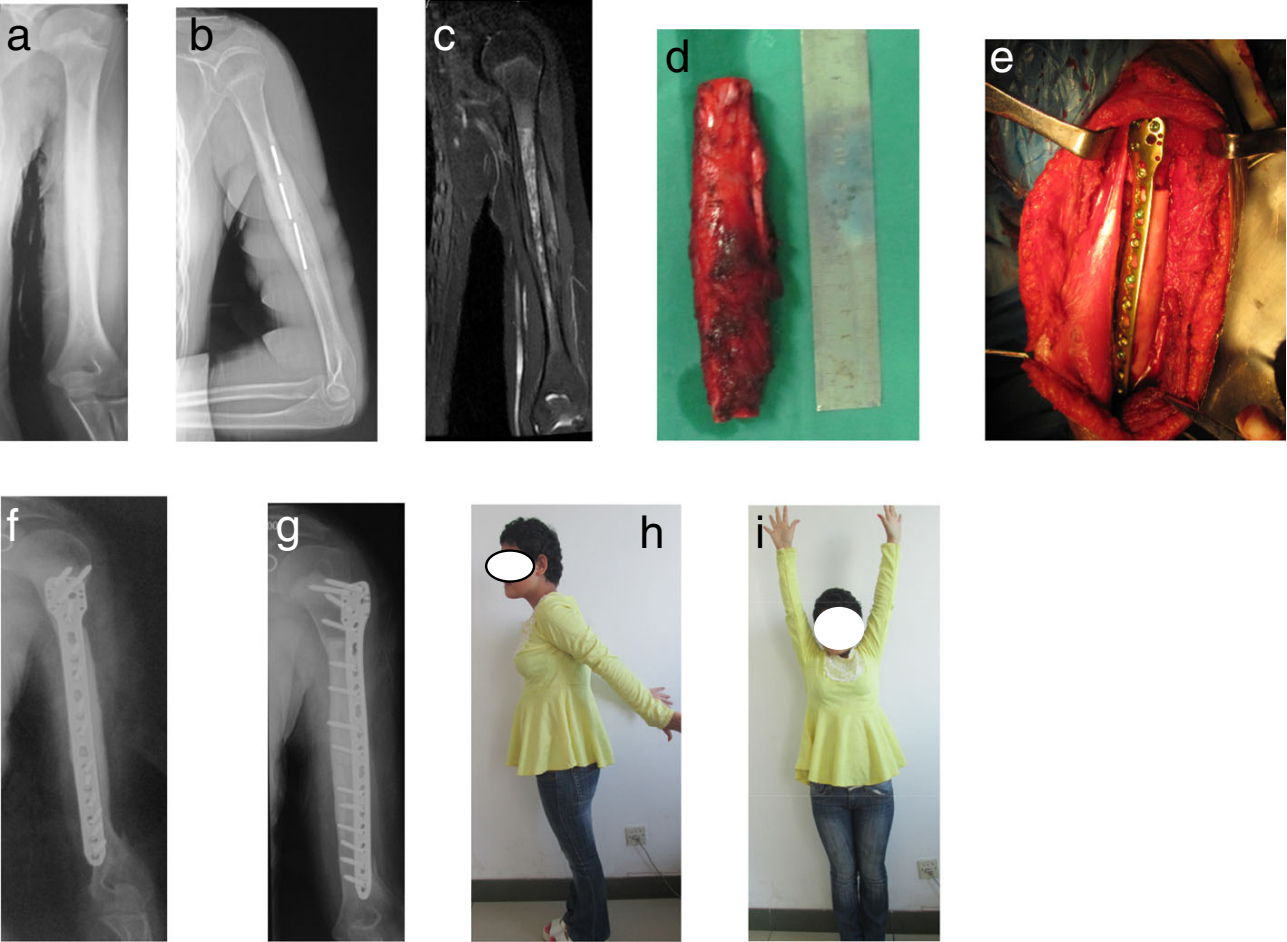
Case 3, a 11-y old female patient with Ewing sarcoma in the diaphysis of left humerus (S5). The preoperative bone strength score is 9. **a, b** The preoperative X-ray showed that osteogenic destruction (**a**) before chemotherapy and obvious calcification (**b**) after chemotherapy. **c** The preoperative MRI showed that abnormal intramedullary high signal with clear borders on T2WI. **d, e** This patient underwent resection of the tumor segment and replantation of the alcohol-inactivated autograft followed by plate internal fixation. **f, g** 5 years after operation,the X-ray indicated that internal fixation was in excellent position with no local tumor recurrence. **h, i** Postoperative shoulder joint function returned to normal range

Extracorporeal devitalized autograft replantation is an effective biological reconstruction method used to repair segmental bone defects following tumor resection of long bones, especially in countries where it is difficult to obtain massive allografts. The advantages of alcohol devitalization are low-cost, easy operation, and excellent shape matching ability [11]. However, there is a trade-off between the thoroughness of tumor devitalization and the loss of bone strength when a tumor segment is inactivated in vitro. We previously reported on the application of alcohol-inactivated autograft replantation for the treatment of a diaphysis humeral Ewing sarcoma patient [18]. Complete bone union was seen at the host-graft interface 5 months after operation, and shoulder and elbow joint function have returned to normal. There were no complications such as fracture of inactivated bone, nonunion, recurrence or lung metastasis that occurred during the follow-up period of more than 5 years (Fig. 3). In our study, 3 patients with primary bone sarcomas of the humeral shaft underwent alcohol-inactivated autograft replantation. They received two courses of neoadjuvant chemotherapy prior to operation. Their response to chemotherapy was relatively satisfactory and preoperative MRI data demonstrated that the tumor did not invade the peripheral blood vessels and nerves. Based on the bone strength scoring system, the average bone strength score of the three cases was 9 (range, 8–10), indicating that the extent of bone destruction was low. Consequently, alcohol inactivated autograft replantation along with joint preservation was performed on the 3 patients. The mean postoperative MSTS score was 28 (28/30, 93.3%) at the end of the follow-up period. Function of the affected limbs had evidently improved by preserving the native shoulder and elbow joint. A retrospective analysis of 191 patients with bone tumors who were treated with the alcohol inactivated autograft replantation showed that the overall local recurrence rate was 26.7%, lung metastasis rate was 27.2%, and bone nonunion rate was 17.3% [19]. One patient suffered local recurrence, which may have been related to inadequate resection margins. The patient died of lung metastasis 28 months after surgery. The average bone healing duration was 6.8 months (range, 6–10 months), which is similar to that of massive allografts [10]. A previous study had showed that the fracture rate of alcohol-inactivated autografts can be as high as 20.4% [19]. In order to reduce the incidence of fracture, we carefully evaluated the mechanical strength of the tumor segments using the bone strength scoring system prior to operation. Reconstruction of bone defects following tumor resection using alcohol-devitalized autografts is not recommended for severe osteolytic lesions with a bone strength score of > 10. No devitalized bone fractures were observed in selected patients.

**Fig. 4:**
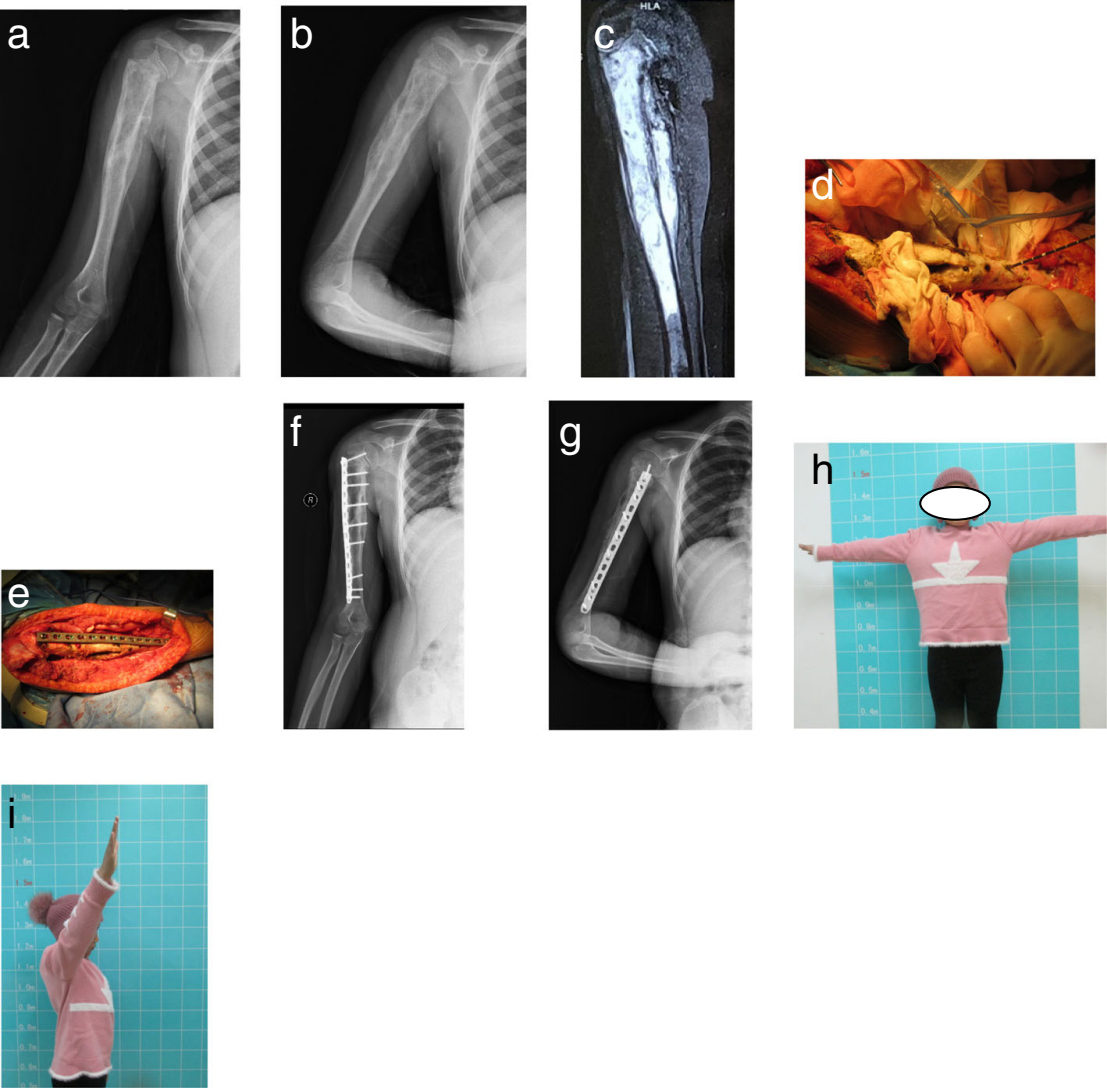
Case 7, a 8-y old female patient with Ewing sarcoma in the right humerus(S45E1). The preoperative bone strength score is 12. **a, b** The post-chemotherapy X-ray showed that obvious calcification after chemotherapy. **c** The post-chemotherapy MRI showed that tumor did not invade epiphyseal plate with a clear bounder. **d, e** This patient underwent in situ microwave ablation and plate internal fixation. **f, g** 15 months after operation,the X-ray indicated that internal fixation was in good position and no local recurrence of tumor occurred. **h, i** The patient received excellent abduction and lifting function of shoulder joint at the last follow-up

In situ microwave ablation was performed on 10 patients with malignant bone tumors in the diaphysis and/or metaphysis part of the humerus. Among them, there were 3 cases of chondrosarcoma, 6 cases of metastatic bone tumors, and 1 case of Ewing sarcoma. Taking the Ewing sarcoma patient as an example, the tumor border was not invaded beyond the epiphyseal plate, and obvious tumor calcification was observed after chemotherapy. This patient underwent in situ microwave ablation and internal fixation. No complications of local recurrence and fracture occurred up to 15 months after surgery (Fig. 4). Fan et al. [20] reported a local recurrence rate of 9.8% in patients with malignant extremity bone tumors treated with microwave inactivation, and the rate of mechanical complications was 2.6%. At the last follow-up visit, the mean MSTS score of the 10 patients was 27 (range, 26–29), which is comparable to values published in existing literature [20]. Fan et al. [20] reported that the over 3-year survival rate of high-grade and low-grade malignancies are 59.1 and 88.7%, respectively. This significant difference indicates that microwave ablation might be more suitable for low-grade malignant bone tumors. Hu et al. [21] found that high cortical brittleness and low biomechanical properties induced by microwave hyperthermia could increase the risk of postoperative pathological fractures. The mean bone strength score of patients who underwent in situ microwave ablation was 9.9 (range, 8–12), indicating that the extent of bone destruction is not severe. Thus, they underwent in situ microwave inactivation and internal fixation. After curettage of inactivated tumor tissue, bone cement was used to fill the tumor cavity and a steel plate was used to strengthen fixation. After operation, the affected extremity was immobilized for 6 weeks. The 10 patients were followed up for 6–60 months, and no postoperative fracture was found. In brief, in situ microwave ablation is suitable for patients with humeral malignancies (low-grade malignant bone tumors or metastatic tumors) in the diaphysis and/or metaphysis with a low bone strength score (≤10).

There are many methods of reconstruction of segmental bone defects from intercalary resection of metastatic tumors, including intercalary prosthesis, massive allografts, intramedullary nail and plate combined with bone cement [22–25]. However, devitalized autografts are not suitable for the reconstruction of metastatic diaphyseal defects with a high bone strength score (> 10). During the early stage of this study, intramedullary nail or plate combined with bone cement was usually used for the reconstruction of segmental defects following the resection of metastatic tumors. The mean postoperative MSTS score of these patients was 25.7 (range, 24–27), indicating 85.7% normal function. One of the six cases suffered from fracture and screw breakage 33 months after operation. Then the patient underwent removal of the plate combined with bone cement and reconstruction using a custom-made intercalary prosthesis (Wego, Beijing, China) (Fig. 1). Some biomechanical studies have demonstrated that intercalary prosthesis performs better in various types of loading (four-point bending, torsion, compression) when compared with other fixation methods [22, 24]. Intercalary prosthetic reconstruction is of benefit to patients with metastatic diaphyseal tumors due to the advantages of immediate stability, preservation of adjacent joints and an early return of function [23, 26]. After 2013, intercalary prosthesis was used to reconstruct segment skeletal defects instead of rush rod or plate augmented with bone cement. The mean postoperative MSTS score of patients who underwent intercalary prosthetic reconstruction was 26.4 (range, 22–29), indicating 88% normal function. However, early loosening has been reported to occur in 9.5–30.8% of cases [27, 28]. Some authors believe that high rotational stress in the upper extremity may lead to early loosening [27, 29]. Among our series of patients, one case of aseptic loosening of the implant with short intramedullary fixation was found 3 months after operation. This may have been related to high rotational stress and traction in the upper limb. Some researchers believe that the length of the remaining bone in prosthesis fixation should be > 5 cm [29, 30]. Some authors have suggested that additional extracortical plates should be added to short-segment intramedullary fixation < 5 cm or bone defects > 10 cm [31, 32]. A biomechanical analysis of a novel intercalary prosthesis for humeral diaphyseal segmental defect reconstruction showed that intercalary prosthesis with plate fixation improves the rigidity of anti-tension and anti-torsion, and diminishes the risk of early loosening and dislocation [33]. In order to reduce the incidence of prosthetic loosening, we used a custom-made intercalary prosthesis and added a PHILOS plate, a straight plate, or a condylar plate for the reconstruction of segmental defects in the proximal, middle, or distal diaphysis humerus, respectively [34].

Wafa et al. [35] reported a mean MSTS score of 24.8 ± 2.6 (range, 18–28) for 28 patients who survived their disease for more than 12 months after total humeral endoprosthetic replacement. In the present study, the mean MSTS score for the 28 patients who underwent joint-sparing limb salvage was 26.6 ± 1.8 (range, 22–30), indicating excellent limb function. All patients had returned to their preoperative daily activities at the last follow-up visit.

There are some limitations and shortcomings in this study. First, the number of cases of different operative procedures is very small due to the infrequent application of joint preservation. Second, this is a single center retrospective study. Up to a certain extent, selection of operative procedures was affected by personal experience and preference. There were also no direct comparisons with other reconstructive methods such as massive allografts or fibular grafts. Third, only a few cases was followed up for more than 5 years.

## Conclusions

Selection of the joint-preserving limb salvage operative procedure should be based on tumor origin, site and bone strength. Alcohol devitalized autograft replantation is applicable for diaphyseal humeral primary malignancies with a good response to chemotherapy and a low bone strength scores (≤10). In situ microwave ablation is suitable for diaphyseal and (or) metaphyseal low-grade malignant bone tumors or metastases with a low bone strength scores (≤10). Intercalary prosthetic reconstruction is preferred for diaphyseal metastases with a high bone strength scores (> 10).

## Data Availability

The datasets generated during and/or analysed during the current study are not publicly available due they are paper document in chinese language store in our hospital archives but are available from the corresponding author on reasonable request.

## Abbreviations

AAR: Alcohol inactivated autograft replantation
MA: Microwave ablation
MRI: Magnetic resonance imaging
MSTS: Musculoskeletal Tumor Society
VAS: Visual analogue scale
